# Multimorbidity characteristics in older adults and their associated factors in complex networks: a cross-sectional study

**DOI:** 10.3389/fpubh.2025.1473572

**Published:** 2025-02-26

**Authors:** Dan Wu, Jiani Xu, Haibo Zhang, Kai Zhang, Yongqian Zhu

**Affiliations:** ^1^Department of Endocrinology, Jiangsu Provincial Official Hospital, Nanjing, Jiangsu, China; ^2^Center for Data Management, The First Affiliated Hospital with Nanjing Medical University, Nanjing, Jiangsu, China; ^3^Jiangsu Province Engineering Research Center of Chronic Disease Big Data Application and Smart Healthcare Service, Nanjing, Jiangsu, China; ^4^Medical Administrative Department, The First Affiliated Hospital with Nanjing Medical University, Nanjing, China; ^5^Pancreas Center, The First Affiliated Hospital with Nanjing Medical University, Nanjing, China; ^6^Pancreas Institution of Nanjing Medical University, Nanjing, China; ^7^Department of Medical Quality Management, The First Affiliated Hospital with Nanjing Medical University, Nanjing, China

**Keywords:** multimorbidity patterns, COVID-19, older adults, inpatients, China

## Abstract

**Background:**

Multimorbidity of chronic diseases has become an increasingly serious public health problem. However, the research on the current situation of multimorbidity in the older adults in Jiangsu, China is relatively lacking.

**Methods:**

We surveyed a total of 229,926 inpatients aged above 60 and with two or more chronic diseases in the First Affiliated Hospital with Nanjing Medical University from January 1, 2015 to December 31, 2021. The Apriori algorithm was used to analyze the association rules of the multimorbidity patterns in old adults.

**Results:**

The mean age of these patients was 72.0 ± 8.7 years, and the male-to-female ratio was 1: 1.53. These patients during the COVID-19 period (from 2020 to 2021) displayed younger, higher male rate, shorter median length of hospital stay, higher ≥6 multimorbidities rate and lower median cost than those not during the COVID-19 period (from 2015 to 2019). In all of these patients, the top 5 chronic diseases were “Hypertensive diseases (I10-I15),” “Other forms of heart disease (I30-I52),” “Diabetes mellitus (E10-E14),” “ischaemic heart diseases (I20-I25)” and “Cerebrovascular diseases (I60-I69).” The complex networks of multimorbidity showed that Hypertensive diseases had a higher probability of co-occurrence with multiple diseases in all these patients, followed by diabetes mellitus, other forms of heart disease, and ischaemic heart diseases (I20-I25).

**Conclusion:**

In conclusion, the patterns of multimorbidity among the aged varied by COVID-19. Our results highlighted the importance of control of hypertensive diseases, diabetes, and heart disease in most periods. However, during the pandemic period, we should pay more attention to diseases that require urgent treatment, such as malignant tumors. For different periods, the spectrum of diseases we focus on should change accordingly.

## Background

Multimorbidity is defined as the coexistence of two or more chronic medical conditions by the World Health Organization (WHO) (1). It includes the co-occurrence of physical diseases such as diabetes, heart disease, and cancer, as well as mental health conditions like depression and anxiety (2). Globally, the prevalence of multimorbidity is increasing significantly. Among US adults with a mean age 48.5 years and 51.0% of women, 8.0% had at least 2 cardiac, renal, and metabolic conditions, and 1.5% had 3 conditions (3). In older adults from US, the prevalence of chronic disease comorbidity was about 77.0% US (4). And approximately 55% of adults aged 65–74 have multimorbidity, while over 70% aged 75 and older in England (5). Similarly, in China, the prevalence of multimorbidity was 52.88% among older adults (≥60 years old) and approximately 30.91% of these older Chinese individuals had at least three comorbidities, specifically 52.91% in females and 47.09% in males (6). Compared with single diseases, multimorbidity is more likely to be associated with aging, more hospital visits, a longer course of treatment, high risk of death, complex care needs, multiple drug use, and excessive medical treatment. Furthermore, patients with more multimorbidity tend to undergo more intensive treatments and poorer overall quality of life (7). Additionally, those patients with three or more multimorbidities typically face 6.6 times higher costs than peers without multimorbidities (8). Multimorbidities in older patients deserve more attention, as China has entered the aging society since 1999, and its aging process is still accelerating. With the rapid change of the disease spectrum, the interweaving of multiple health factors, and the accelerating process of population aging, it has become an important task for us to promote a healthy aging strategy, grasp the pattern of disease development, and identify the health needs of the aging population. Especially, whether there will be changes in the spectrum of multimorbidities during a pandemic infectious disease outbreak is still unknown. To address the challenges posed by multimorbidity, we should gain a better understanding of the current distribution of multimorbidity, which is crucial to have prior knowledge of its prevalence and patterns. Furthermore, policymakers and healthcare providers must also adopt measures to support the care and treatment of patients with multimorbidity, ensuring they receive high-quality medical services. In this study, we aim to observe the patterns of multimorbidities during the pandemic infectious diseases (COVID-19) as well as during non-pandemic periods.

## Methods

### Materials and methods

To reveal the current situation of multimorbidity in the older adults in China, we took hospitalized aging patients with multimorbidity as the research objects. This study was approved by the Institutional Review Board of the First Affiliated Hospital with Nanjing Medical University (2021-SR-163). Data collected retrospectively did not require informed consent. And all the data was from consecutively enrolled inpatients in the First Affiliated Hospital with Nanjing Medical University from January 1, 2015 to December 31, 2021. The enrollment strategy for patients was as follows. Inclusion criteria: (1) age ≥ 60 years old; (2) suffering from two or more diseases; (3) length of hospital stay ≥1 day. Exclusion criteria: (1) absence of clinical data; (2) missing the International Classification of Diseases (ICD) code for the disease. Diseases were categorized according to the ICD (10th vision). All the ICD-10 chapter except O00 to U99 was used for categorization (e.g., certain infectious and parasitic diseases, Neoplasms; Endocrine nutritional and metabolic diseases; etc.). A total of 229,926 records of data were collected. Demographic (age, gender) and clinical data (date of discharge, length of stay, cost) were collected from the electronic medical record system. All patients with multimorbidity, are defined as the simultaneous presence of at least two diseases from ICD-10.

### Statistical analysis

As a retrospective study and the purpose of this study, the sample size was not estimated prior. However, a sample size greater than 10,000 is necessary. Age was described with mean and standard deviation (SD) and compared by *t*-test. Length of stay, number of multimorbidity, and cost was described as median and interquartile range (IQR; 25th-75th percentiles) and compared by Wilcoxon’s rank sum test. And gender was described as counts and percentages and compared by Chi-square test or Fisher exact test, as appropriate.

The association rules of multimorbidities in patients were analyzed by the Apriori algorithm using the “*arules*” package and visualized graphs by the “*arulesViz*” package. The Apriori algorithm is a classic approach for association rule mining, primarily used to uncover interesting relationships between items in large datasets. The resulting association rules are expressed as “Support,” “Confidence,” and “Lift.” These three parameters are standard metrics used to measure the association between items (herbs) in the algorithm. “Support” measures the frequency of an item set in the dataset. “Confidence” measures the trustworthiness of an association rule. And “Lift” measures the strength of the association between A and B compared to the expected support if A and B were chosen randomly. The bound value of Lift was 1. When the Lift >1, it suggested that there is a positive correlation between disease A and disease B and that there is multiple possibility of the occurrence of disease B under the condition of disease A. In our study, the screening conditions of association rules were set as follows: the min-support was 5.0%, the min-confidence was 10.0%, and the left-hand-side was 2, to explore the correlation between a variety of diseases. A two-sided *p* < 0.05 was considered statistically significant. All analyses were performed using R (version: 4.1.1).

## Results

**Figure 1:**
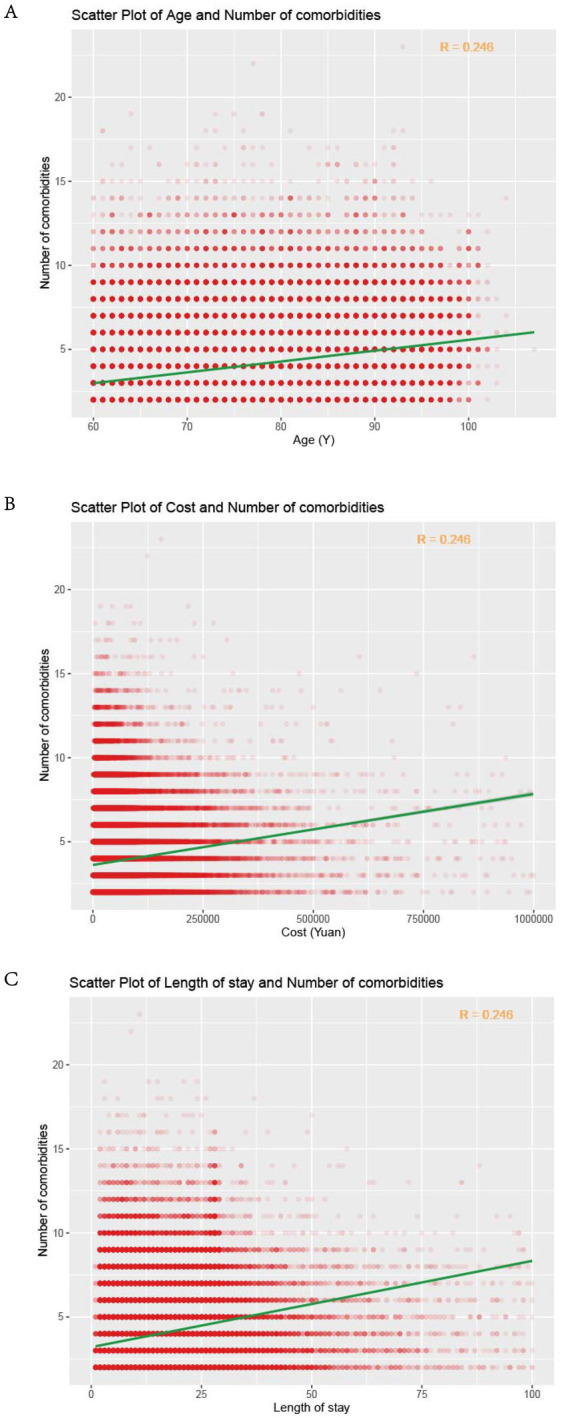
Correlation analysis of the number of multimorbidities and age **(A)**, cost **(B)** and length of stay **(C)** in hospital. The red dots represented individual cases, the green line was the fitted line of linear correlation, and the R in the top right corner represented the correlation coefficient.

Overall, 229,926 consecutive patients with two or more multimorbidities (mean age years; Female: Male ratio 1.53:1) were enrolled in this study including 864,899 medical records. The mean age was 72.0 ± 8.7 years, and 60.5% of those with specific gender information were female. The comparison between patients before the COVID-19 period (from 2015 to 2019, *n* = 150,035) and during the COVID-19 period (from 2020 to 2021, *n* = 79,891) was unbalanced (Table 1). In brief, patients during the pandemic period displayed younger (71.5 ± 8.4 vs. 72.3 ± 8.9 years), higher male rate (61.64% vs. 59.89%), the shorter median length of hospital stay (7 vs. 8 days), higher ≥6 multimorbidity rate (18.30% vs. 16.08%) and lower median cost (16,402 vs. 18,475) than non-pandemic period. Then we compared the five high-frequency multimorbidity spectra in these two periods, and the results showed that the multimorbidity spectra were different in the two different periods (*p* < 0.0001). The incidence of malignant tumors increased during the epidemic period, and these patients had to seek medical treatment, while the proportion of some chronic diseases such as hypertensive diseases and diabetes mellitus decreased (Table 1). The positive correlation between the number of multimorbidities and age, hospitalization cost, length of stay suggests that these patients with more multimorbidities require more time and money for treatment, which also increases their economic and social burdens (Figure 1).

**Table 1:** Basic information on inpatient older adult patients, 2015–2021.

	Total	2015 ~ 2019	2020 ~ 2021	Statistics	*p*-value
Number	229,926	150,035	79,891		
Age (Y)	72.0 ± 8.7	72.3 ± 8.9	71.5 ± 8.4	22.917^†^	<0.0001^†^
Gender (%)				66.72^‡^	<0.0001^‡^
Male	139,090 (39.51)	89,849 (59.89)	49,241 (61.64)		
Female	90,836 (39.51)	60,186 (40.11)	30,650 (38.36)		
Length of hospital stay (day, IQR)	8 (5, 14)	8 (5, 14)	7 (4, 12)	*Z* = 62.961^§^	<0.0001^§^
Number of co-morbidities (%)				284.9^§^	<0.0001^§^
2	73,983 (32.18)	49,642 (33.09)	24,341 (30.47)		
3	55,428 (24.11)	36,415 (24.27)	19,013 (23.80)		
4	37,660 (16.38)	24,354 (16.23)	13,306 (16.66)		
5	24,113 (10.49)	15,505 (10.33)	8,608 (10.77)		
≥6	38,742 (16.85)	24,119 (16.08)	14,623 (18.30)		
Major co-morbidities (%)				1.3×10^4^	<0.0001^‡^
Hypertensive diseases, I10-I15	44,678 (19.43)	30,573 (20.38)	14,105 (17.66)		
Diabetes mellitus, E10-E14	20,298 (8.83)	14,142 (9.43)	6,156 (7.71)		
Ischaemic heart diseases, I20-I25	16,517 (7.18)	11,788 (7.86)	4,729 (5.92)		
Cerebrovascular diseases, I60-I69	14,229 (6.19)	9,865 (6.58)	4,364 (5.46)		
Malignant neoplasms, C00-C97	11,599 (5.04)	1886 (1.26)	9,713 (12.16)		
Cost (Yuan, IQR)	17,698 (9,483, 39,907)	18,475 (9,671, 41,931)	16,402 (9,183, 35,969)	*Z* = 19.916^§^	<0.0001^§^

^†Student’s t-test was used to assess the difference of age between different period.^

^‡Chi2 test was used to assess the percentages of gender between different period.^

^§Wilcoxon’s rank sum test was performed to investigate the discrepancy between length of stay, number of multimorbidity, and cost.^

**Figure 2:**
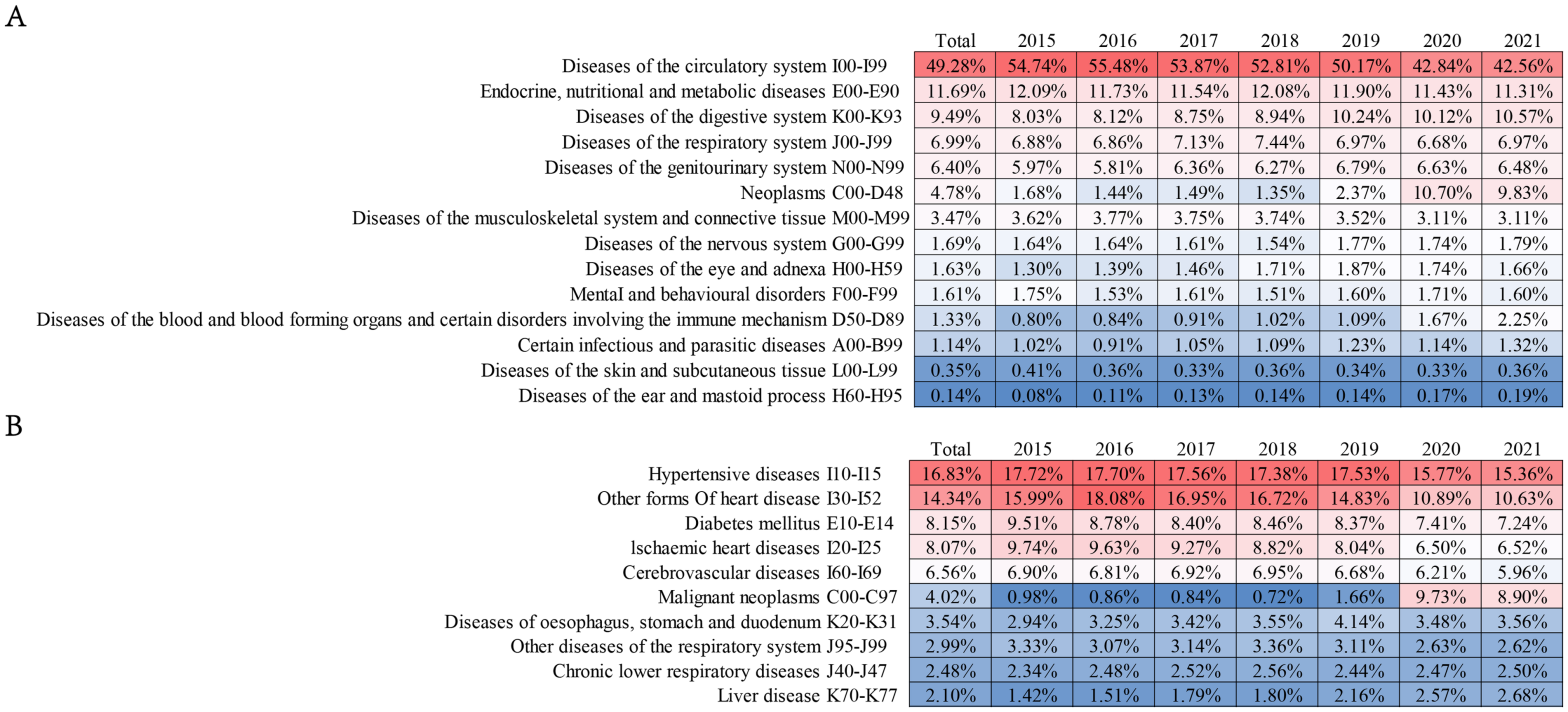
The frequency of various comorbidities in older adults. Panel **(A)** was for ICD-10 categories and **(B)** for ICD-10 subcategories. Each column represented a year (from 2015 to 2021), with red indicating a high incidence rate (the darker the red, the higher the rate), and blue indicating a low incidence rate (the darker the blue, the lower the rate). The percentage value in each cell represented the percentage occurrence of that particular comorbidity for that year.

According to the ICD-10 categories, the top 5 most common diseases in hospitalized older adults multimorbidities were diseases of the circulatory system (I00-I99, 49.28%), endocrine, nutritional, and metabolic diseases (E00-E90, 11.69%), diseases of the digestive system (K00-K93, 9.49%), diseases of the respiratory system (J00-J99, 6.99%) and diseases of the genitourinary system (N00-N99, 6.40%). Although the highest number of diseases of the circulatory system was recorded, the percentage declined by over 10% from 2015 to 2021. In contrast, the percentage of neoplasms was only 1.68% in 2015 but exceeded 10% in both 2020 and 2021 (Figure 2A). For ICD-10 subcategories, the detailed disease prevalence was shown in Figure 2B. The top 5 diseases were “Hypertensive diseases (I10-I15),” “Other forms of heart disease (I30-I52),” “Diabetes mellitus (E10-E14),” “Ischaemic heart diseases (I20-I25)” and “Cerebrovascular diseases (I60-I69),” with average prevalence rates of 16.83, 14.34, 8.15, 8.07 and 6.56%. Based on the proportion of malignant neoplasms increased to over 10% after 2020, it appears that there has been a shift in attention toward certain diseases, as evidenced by the increasing percentage of neoplasms and decreasing percentage of circulatory diseases.

**Figure 3:**
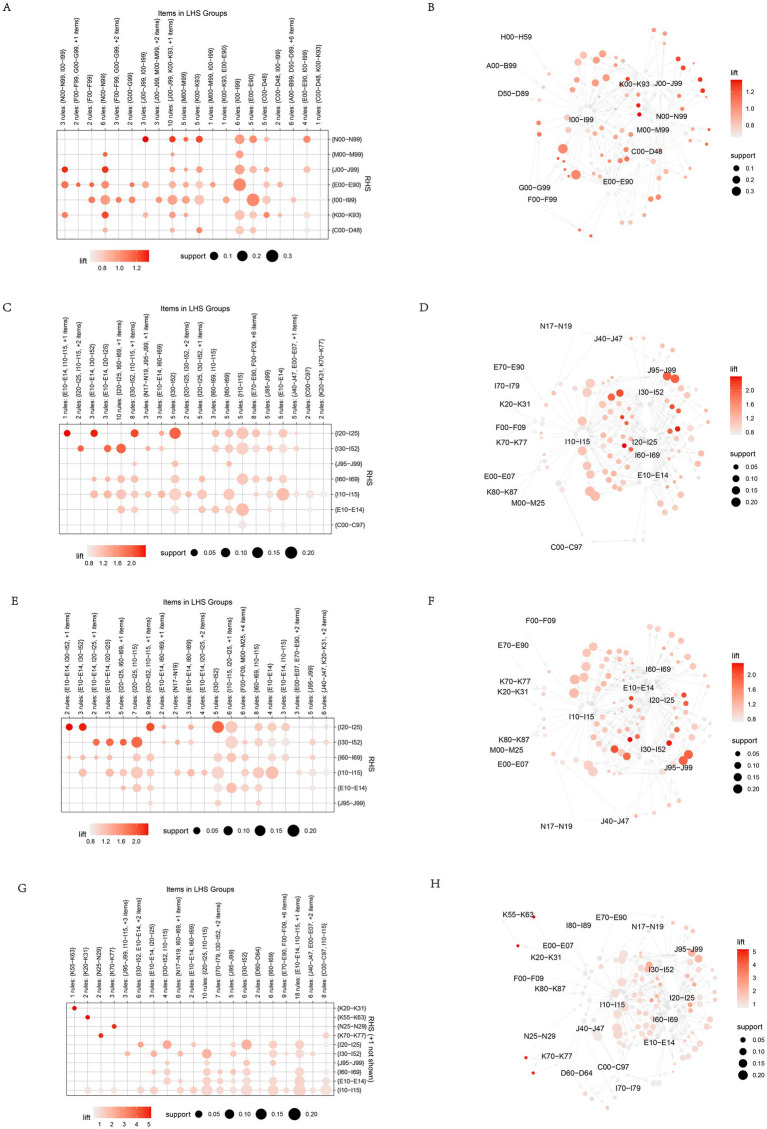
Relationship diagrams of multimorbidity network for rules. **(A,B)** Association rule analysis of multimorbidities pairs in older adults with multimorbidity from 2015 to 2021 (Based on ICD-10 categories). **(C,D)** Association rule analysis of multimorbidities pairs in older adults with multimorbidity from 2015 to 2021 (based on ICD-10 subcategories). **(E,F)** Association rule analysis of multimorbidities pairs in older adults with multimorbidity from 2015 to 2019 (Based on ICD-10 subcategories). **(G,H)** Association rule analysis of multimorbidities pairs in older adults with multimorbidity from 2020 to 2021 (Based on ICD-10 subcategories). On the left side of the figure were the associated graphs, with rows and columns representing different diseases, respectively. The size of the intersection points indicates the magnitude of support, while the shade of color represents the magnitude of lift. On the right side of the image was the visualization of association rules, where the size of the points also depicted the magnitude of support, and the shade of color represents the magnitude of lift.

The complex networks of multimorbidity suffered by older adult patients were visualized through relationship diagrams, which were useful to take a holistic view of the relationship between diseases and their change patterns (Figure 3). According to the Apriori algorithm, the top 10 frequent relationships were shown in Table 2. Hypertensive diseases (I10-I15) had a higher probability of co-occurrence with multiple diseases in all these patients, including diabetes mellitus (E10-E14). Other forms of heart disease (I30-I52), ischaemic heart diseases (I20-I25), and Cerebrovascular diseases (I60-I69) with support 0.222, 0.201, 0.185 and 0.162 the rule of other forms of heart disease (I30-I52) and hypertensive diseases (I10-I15) was newly surfaced during the COVID-19 period (Table 2 and Figures 3C,D). In patients from 2015 to 2019, the top five rules were diabetes mellitus (E10-E14) and hypertensive diseases (I10-I15), ischaemic heart diseases (I20-I25) and hypertensive diseases (I10-I15), ischaemic heart diseases (I20-I25) and other forms of heart disease (I30-I52), cerebrovascular diseases (I60-I69) and hypertensive diseases (I10-I15), and ischaemic heart diseases (I20-I25) + other forms of heart disease (I30-I52) and hypertensive diseases (I10-I15) (Table 2 and Figures 3E,F), while diabetes mellitus (E10-E14) and hypertensive diseases (I10-I15), ischaemic heart diseases (I20-I25) and hypertensive diseases (I10-I15), other forms of heart disease (I30-I52) and hypertensive diseases (I10-I15), Cerebrovascular diseases (I60-I69) and hypertensive diseases (I10-I15), and ischaemic heart diseases (I20-I25) and other forms of heart disease (I30-I52) in patients from 2020 to 2021 (Table 2 and Figures 3G,H). The results of chronic disease comorbidity pattern of the older adults show that among the top 10 chronic disease comorbidity combinations, “hypertension + diabetes” is the chronic disease comorbidity pattern with the highest prevalence rate, followed by hypertension and heart diseases (other forms of heart disease or ischaemic heart diseases). In addition, different types of heart diseases also occur at the same time. From the perspective of network influence, hypertension, diabetes, ischemic heart disease and other forms of heart disease are the four most common core chronic diseases, and they have the most close interaction and connection with other chronic diseases. According to this older adult patients’ multimorbidity networks, visualized through diagrams, is showed that hypertensive diseases was commonly co-occurring with diabetes, ischaemic heart diseases, and cerebrovascular diseases, with consistent patterns pre-and post-COVID-19.

**Table 2:** Association rule analysis of multimorbidities.

Lhs		Rhs	Support^¶^	Coverage^¶^	Lift^¶^
2015 ~ 2021					
Diabetes mellitus, E10-E1	=>	Hypertensive diseases, I10-I15	0.222	0.294	1.194
Other forms of heart disease, I30-I52	=>	Hypertensive diseases, I10-I15	0.201	0.315	1.012
Ischaemic heart diseases, I20-I25	=>	Hypertensive diseases, I10-I15	0.185	0.264	1.109
Ischaemic heart diseases, I20-I25	=>	Other forms of heart disease, I30-I52	0.164	0.264	1.97
Cerebrovascular diseases, I60-I69	=>	Hypertensive diseases, I10-I15	0.162	0.232	1.102
Ischaemic heart diseases, I20-I25; Other forms of heart disease, I30-I52	=>	Hypertensive diseases, I10-I15	0.112	0.164	1.082
Hypertensive diseases, I10-I15; I20-I25	=>	Other forms of heart disease, I30-I52	0.112	0.185	1.922
Hypertensive diseases, I10-I15; Other forms of heart disease, I30-I52	=>	Ischaemic heart diseases, I20-I25	0.112	0.201	2.106
Ischaemic heart diseases, I20-I25	=>	Diabetes mellitus, E10-E14	0.085	0.264	1.093
Cerebrovascular diseases, I60-I69	=>	Other forms of heart disease, I30-I52	0.079	0.232	1.073
2015 ~ 2019					
Diabetes mellitus, E10-E14	=>	Hypertensive diseases, I10-I15	0.23	0.302	1.18
Ischaemic heart diseases, I20-I25	=>	Hypertensive diseases, I10-I15	0.198	0.283	1.082
Ischaemic heart diseases, I20-I25	=>	Other forms of heart disease, I30-I52	0.188	0.283	1.899
Cerebrovascular diseases, I60-I69	=>	Hypertensive diseases, I10-I15	0.169	0.241	1.084
Ischaemic heart diseases, I20-I25; Other forms of heart disease, I30-I52	=>	Hypertensive diseases, I10-I15	0.127	0.188	1.05
Hypertensive diseases, I10-I15; Ischaemic heart diseases, I20-I25	=>	Other forms of heart disease, I30-I52	0.127	0.198	1.843
Hypertensive diseases, I10-I15; Other forms of heart disease, I30-I52	=>	Ischaemic heart diseases, I20-I25	0.127	0.223	2.016
Diabetes mellitus, E10-E14	=>	Ischaemic heart diseases, I20-I25	0.089	0.302	1.049
Cerebrovascular diseases, I60-I69	=>	Other forms of heart disease, I30-I52	0.086	0.241	1.024
Cerebrovascular diseases, I60-I69	=>	Ischaemic heart diseases, I20-I25	0.075	0.241	1.094
2020 ~ 2021					
Diabetes mellitus, E10-E14	=>	Hypertensive diseases, I10-I15	0.206	0.281	1.219
Ischaemic heart diseases, I20-I25	=>	Hypertensive diseases, I10-I15	0.161	0.23	1.163
Other forms of heart disease, I30-I52	=>	Hypertensive diseases, I10-I15	0.161	0.253	1.056
Cerebrovascular diseases, I60-I69	=>	Hypertensive diseases, I10-I15	0.148	0.216	1.139
Ischaemic heart diseases, I20-I25	=>	Other forms of heart disease, I30-I52	0.121	0.23	2.084
Ischaemic heart diseases, I20-I25; Other forms of heart disease, I30-I52	=>	Hypertensive diseases, I10-I15	0.084	0.121	1.151
Hypertensive diseases, I10-I15; Other forms of heart disease, I30-I52	=>	Ischaemic heart diseases, I20-I25	0.084	0.161	2.271
Ischaemic heart diseases, I20-I25	=>	Diabetes mellitus, E10-E14	0.077	0.23	1.191
Malignant neoplasms, C00-C97	=>	Diabetes mellitus, E10-E14	0.071	0.314	0.8
Cerebrovascular diseases, I60-I69	=>	Diabetes mellitus, E10-E14	0.067	0.216	1.109

^¶Apriori algorithm was used to assess the association rules of multimorbidities in inpatients. “Support,” “Confidence” are standard metrics used to measure the association between items. A two-sided p < 0.05 was considered statistically significant.^

## Discussion

With the increase in life expectancy all over the world, multimorbidity has become a more noticeable public health problem, causing its prevalence was positively correlated with age and it’s associated with poorer life quality, premature death, and an increased need for health care (9–11). Our study, among more than 200,000 hospitalized older adults, revealed that circulatory system diseases, especially hypertension and ischemic heart disease, were the most common conditions in multimorbidities. And many studies have suggested that hypertension and heart disease are the most common chronic diseases (12–14). These diseases were often accompanied by endocrine, nutritional, and metabolic disorders, particularly diabetes. Besides, compared with 2015–2019, the prevalence of neoplasms in multimorbidity has increased significantly in 2020–2021, which prompts that compared to some less serious multimorbidities, such as hypertension, the diagnosis and treatment of tumors are more urgent during the pandemic. We need to pay more attention to the public health and clinical research significance of neoplasms in multimorbidity. COVID-19 had a great impact on many social aspects, especially the attention given to different diseases (15, 16). It would also have a great impact on the comorbidity spectrum of hospital inpatients. On the one hand, it might indeed be a change in the disease incidence. On the other hand, some patients with chronic diseases choose not to seek medical treatment due to the fear of spreading COVID-19 or other reasons.

Cardiometabolic diseases as one of the most frequent multimorbidity groups pose a great threat to the life and health of the aged, which is consistent with previous studies (17–19). Cardiovascular aging and metabolic decline with age are both continuous and irreversible processes that may be facilitated by genetics and poor lifestyle habits (20). Hypertensive disease is a prominent health issue, not only as a risk factor for cardiovascular morbidity and mortality (21) but also increases the incidence of trauma in aging people (22). Over the course of this century, the prevalence of hypertension and average blood pressure has declined in high-income regions but increased in less-developed regions. In 2015, 88% of the 8.5 million hypertension-related deaths worldwide occurred in less-developed regions. The onset of hypertension in our country is thought to be related to patients eating too much salt, and we believe that our country still has a lot of work to do in terms of a healthy diet and active health (23).

Diabetes mellitus is a chronic metabolic disease characterized by elevated fasting blood glucose, which can affect both small and large blood vessels and the most prevalent complications are kidney disease, blindness, and amputations (24). The high economic burden of diabetes also makes the disease an important clinical and public health problem, as shown in the diabetes-related Cost of Disease (COI) study (25). Fortunately, due to the continuous improvement of China’s medical insurance policy and the implementation of the national organization of centralized drug procurement measures, the hospitalization expenditure of older adults with multimorbidity has decreased in 2020–2022 compared with 2015–2019, which indicates that these measures are of great significance to effectively reduce the economic burden of patients.

As mentioned above, the incidence of neoplasms in multimorbidity has increased significantly in recent years. It might be related to global aging. In 2012, 6.7 million new cancer cases (about half of all cancers) were diagnosed among the aged worldwide, and nearly 48% of these cases occurred in low-and middle-income countries (26). Meanwhile, it was also affected by COVID-19, which might cause certain difficulties in the treatment of some diseases (27). In addition, epidemiological studies have found that diabetes is associated with increased incidence and mortality of a variety of tumors, including colorectal cancer and breast cancer (28). This is related to various molecular mechanisms such as hyperglycemia activates cancer-related signaling pathways (29). It suggested that primary health care institutions could effectively reduce the incidence of chronic disease comorbidity in the older adults or delay its progression by preventing and treating hypertension and improving the efficiency of hypertension management, ultimately improving the physical function and quality of life of the older adults.

With the accelerating process of aging society, comorbidity has become one of the most important health problems faced by the older adults and gradually attracted widespread attention from the academic community. There are complex associations among comorbidities, which combined with the differences of individual characteristics, further lead to the complexity and heterogeneity of multimorbidities of health management. This study attempts to present the comorbidity relationship and its disease burden from a holistic and systematic perspective through association analysis, to provide a basis for the study of senile comorbidity. Given the complexity and heterogeneity of senile multimorbidities, we have some suggestions. Firstly, we should strengthen multidisciplinary cooperation, such as epidemiology, internal medicine, surgery, oncology, etc., establish multidisciplinary multimorbidities research teams, and utilize their respective advantages to carry out comprehensive research. Secondly, we can establish a large sample, multi-center comorbidity database, to collect and collate comorbidity research data and clinical practice experience, and provide more accurate and detailed information for comorbidity research. Thirdly, we can promote and apply advanced technology, such as smart wearable medical devices, gene sequencing, bioinformatics, and so on, in combination with traditional research methods, to carry out in-depth and efficient comorbid research. Moreover, the accuracy, repeatability, and interpretability of comorbidity studies can be improved using multi-layered improved research design methods, while minimizing potential bias. Researchers can apply a variety of research methods, such as cohort study, case–control study, longitudinal study, etc., to analyze the mechanism and influencing factors of comorbidity from different perspectives, and carry out comprehensive and in-depth research on comorbidity.

However, there are some limitations in this study. This is a retrospective study and there may be some unknown biases. The detailed information, such as disease severity and income, was not included in this study due to data availability. Therefore, further research is needed using a prospective study design for older adults. In addition, our study is limited to a single hospital and may not be able to effectively evaluate the impact of demographic and spatial factors, which may be problematic to generalize to regions with different development levels in China. A multi-center study should be performed to further analyze and explain the demographic and spatial distribution of multimorbidity.

## Conclusion

With the gradual advance of aging, the number of patients with multimorbidity will gradually increase. How to reduce the number of multimorbidity and cases with multimorbidity under the new medical environment and meet their increasing personalized health needs has become an urgent challenge. Meanwhile, the changes in the comorbidity spectrum that occur during infectious pandemics also deserve our attention. In most periods, Hypertensive diseases is the highest occurrence of comorbidities, followed by diabetes mellitus. These are the two most noteworthy chronic diseases. However, during the pandemic period, the proportion of medical visits for some diseases that must be treated, such as malignant tumors, will increase, and more attention will be paid to some diseases that must be treated during this period. Better attention to the rules of occurrence among comorbidities can help to allocate medical resources more rationally.

## Data Availability

The original contributions presented in the study are included in the article/supplementary material, further inquiries can be directed to the corresponding author.

## References

[1] Organization, W.H. The world health report 2008: primary health care now more than ever, vol. 148. The World Health Organization (WHO) (2008).

[2] Barnett K Mercer SW Norbury M Watt G Wyke S Guthrie B. Epidemiology of multimorbidity and implications for health care, research, and medical education: a cross-sectional study. Lancet. (2012) 380:37–43. doi: 10.1016/S0140-6736(12)60240-2, PMID: 22579043

[3] Ostrominski JW Arnold SV Butler J Fonarow GC Hirsch JS Palli SR. Prevalence and overlap of cardiac, renal, and metabolic conditions in US adults, 1999–2020. JAMA Cardiol. (2023) 8:1050–60. doi: 10.1001/jamacardio.2023.3241, PMID: 37755728 PMC10535010

[4] Ornstein SM Nietert PJ Jenkins RG Litvin CB. The prevalence of chronic diseases and multimorbidity in primary care practice: a PPRNet report. J Am Board Fam Med. (2013) 26:518–24. doi: 10.3122/jabfm.2013.05.130012, PMID: 24004703

[5] Cassell A Edwards D Harshfield A Rhodes K Brimicombe J Payne R. The epidemiology of multimorbidity in primary care: a retrospective cohort study. Br J Gen Pract. (2018) 68:e245–51. doi: 10.3399/bjgp18X695465, PMID: 29530918 PMC5863678

[6] Zhao M He X Li T Shao H Huo Q Li Y. Early-life factors and multimorbidity risk later in older age: evidence based on CHARLS. Gerontology. (2023) 69:1347–57. doi: 10.1159/000532060, PMID: 37725916

[7] Kristensen T Olsen KR Schroll H Thomsen JL Halling A. Association between fee-for-service expenditures and morbidity burden in primary care. Eur J Health Econ. (2014) 15:599–610. doi: 10.1007/s10198-013-0499-7, PMID: 23818280

[8] McPhail SM. Multimorbidity in chronic disease: impact on health care resources and costs. Risk Manag Healthc Policy. (2016) 9:143–56. doi: 10.2147/RMHP.S97248, PMID: 27462182 PMC4939994

[9] Ben HC Fayosse A Landre B Raggi M Bloomberg M Sabia S. Association between age at onset of multimorbidity and incidence of dementia: 30 year follow-up in Whitehall II prospective cohort study. BMJ. (2022) 376:e068005. doi: 10.1136/bmj-2021-068005, PMID: 35110302 PMC9086721

[10] Marengoni A Angleman S Melis R Mangialasche F Karp A Garmen A. Aging with multimorbidity: a systematic review of the literature. Ageing Res Rev. (2011) 10:430–9. doi: 10.1016/j.arr.2011.03.003, PMID: 21402176

[11] Castro-de-Araujo L Cortes F de Siqueira FN Rodrigues E Machado DB de Araujo J. Patterns of multimorbidity and some psychiatric disorders: a systematic review of the literature. Front Psychol. (2022) 13:940978. doi: 10.3389/fpsyg.2022.940978, PMID: 36186392 PMC9524392

[12] Alamnia TT Sargent GM Kelly M. Patterns of non-communicable disease, multimorbidity, and population awareness in Bahir Dar, Northwest Ethiopia: a cross-sectional study. Int J Gen Med. (2023) 16:3013–31. doi: 10.2147/IJGM.S421749, PMID: 37465551 PMC10351527

[13] Franken DL Dias-da-Costa JS Olinto M Sturmer J Bordin RB Paniz V. Multimorbidity patterns: obesity as the main modifiable risk factor in adult women in southern Brazil. Arch Endocrinol Metab. (2023) 67:e000642. doi: 10.20945/2359-3997000000642, PMID: 37249464 PMC10665044

[14] Zhao X Zhang Q Ma C Liu H Chen Y. Association between multimorbidity patterns and healthcare costs among middle-aged and older adults in China. Arch Gerontol Geriatr. (2023) 109:104959. doi: 10.1016/j.archger.2023.104959, PMID: 36804649

[15] Smith BJ Lim MH. How the COVID-19 pandemic is focusing attention on loneliness and social isolation. Public Health Res Pract. (2020) 30:2008. doi: 10.17061/phrp3022008, PMID: 32601651

[16] Blazquez-Fernandez C Lanza-Leon P Cantarero-Prieto D. A systematic review on suicide because of social isolation/and loneliness: does COVID-19 make a difference? J Public Health. (2023) 45:680–8. doi: 10.1093/pubmed/fdad001, PMID: 36680431

[17] Yao SS Cao GY Han L Chen ZS Huang ZT Gong P. Prevalence and patterns of multimorbidity in a nationally representative sample of older Chinese: results from the China health and retirement longitudinal study. J Gerontol A Biol Sci Med Sci. (2020) 75:1974–80. doi: 10.1093/gerona/glz185 31406983

[18] Rodrigues LP Vissoci J Franca DG Caruzzo NM Batista S de Oliveira C. Multimorbidity patterns and hospitalisation occurrence in adults and older adults aged 50 years or over. Sci Rep. (2022) 12:11643. doi: 10.1038/s41598-022-15723-4, PMID: 35804008 PMC9270321

[19] Fan J Sun Z Yu C Guo Y Pei P Yang L. Multimorbidity patterns and association with mortality in 0.5 million Chinese adults. Chin Med J. (2022) 135:648–57. doi: 10.1097/CM9.0000000000001985, PMID: 35191418 PMC9276333

[20] Freisling H Viallon V Lennon H Bagnardi V Ricci C Butterworth AS. Lifestyle factors and risk of multimorbidity of cancer and cardiometabolic diseases: a multinational cohort study. BMC Med. (2020) 18:5. doi: 10.1186/s12916-019-1474-7, PMID: 31918762 PMC6953215

[21] Pirzada A Cai J Cordero C Gallo LC Isasi CR Kunz J. Risk factors for cardiovascular disease: Knowledge gained from the Hispanic community health study/study of Latinos. Curr Atheroscler Rep. (2023) 25:785–93. doi: 10.1007/s11883-023-01152-9 37773246 PMC12344663

[22] Bromfield SG Ngameni CA Colantonio LD Bowling CB Shimbo D Reynolds K. Blood pressure, antihypertensive polypharmacy, frailty, and risk for serious fall injuries among older treated adults with hypertension. Hypertension. (2017) 70:259–66. doi: 10.1161/HYPERTENSIONAHA.116.09390, PMID: 28652459 PMC5667360

[23] Zhou B Perel P Mensah GA Ezzati M. Global epidemiology, health burden and effective interventions for elevated blood pressure and hypertension. Nat Rev Cardiol. (2021) 18:785–802. doi: 10.1038/s41569-021-00559-8, PMID: 34050340 PMC8162166

[24] Forbes JM Cooper ME. Mechanisms of diabetic complications. Physiol Rev. (2013) 93:137–88. doi: 10.1152/physrev.00045.2011 23303908

[25] Ng CS Lee JY Toh MP Ko Y. Cost-of-illness studies of diabetes mellitus: a systematic review. Diabetes Res Clin Pract. (2014) 105:151–63. doi: 10.1016/j.diabres.2014.03.020, PMID: 24814877

[26] Pilleron S Sarfati D Janssen-Heijnen M Vignat J Ferlay J Bray F. Global cancer incidence in older adults, 2012 and 2035: a population-based study. Int J Cancer. (2019) 144:49–58. doi: 10.1002/ijc.31664, PMID: 29978474

[27] Onyeaka H Anumudu CK Al-Sharify ZT Egele-Godswill E Mbaegbu P. COVID-19 pandemic: a review of the global lockdown and its far-reaching effects. Sci Prog. (2021) 104:368504211019854. doi: 10.1177/00368504211019854, PMID: 34061685 PMC10454957

[28] Pearson-Stuttard J Papadimitriou N Markozannes G Cividini S Kakourou A Gill D. Type 2 diabetes and Cancer: an umbrella review of observational and Mendelian randomization studies. Cancer Epidemiol Biomarkers Prev. (2021) 30:1218–28. doi: 10.1158/1055-9965.EPI-20-1245, PMID: 33737302 PMC9398112

[29] Supabphol S Seubwai W Wongkham S Saengboonmee C. High glucose: an emerging association between diabetes mellitus and cancer progression. J Mol Med (Berl). (2021) 99:1175–93. doi: 10.1007/s00109-021-02096-w, PMID: 34036430

